# Recent Advances in High‐Rate Solar‐Driven Interfacial Evaporation

**DOI:** 10.1002/advs.202401322

**Published:** 2024-05-05

**Authors:** Hyeon Tae Kim, Ligy Philip, Andrew McDonagh, Md Johir, Jiawei Ren, Ho Kyong Shon, Leonard D. Tijing

**Affiliations:** ^1^ Centre for Technology in Water and Wastewater School of Civil and Environmental Engineering University of Technology Sydney PO Box 123, 15 Broadway Ultimo NSW 2007 Australia; ^2^ ARC Research Hub for Nutrients in a Circular Economy University of Technology Sydney PO Box 123, 15 Broadway Ultimo NSW 2007 Australia; ^3^ Environmental Engineering Division Department of Civil Engineering IIT Madras Chennai 600 036 India; ^4^ School of Mathematical and Physical Sciences University of Technology Sydney 15 Broadway Ultimo NSW 2007 Australia; ^5^ Faculty of Architecture, Civil and Transportation Engineering Beijing University of Technology Beijing 100124 P. R. China

**Keywords:** desalination, high evaporation, solar‐driven interfacial evaporation, water purification

## Abstract

Recent advances in solar‐driven interfacial evaporation (SDIE) have led to high evaporation rates that open promising avenues for practical utilization in freshwater production and industrial application for pollutant and nutrient concentration, and resource recovery. Breakthroughs in overcoming the theoretical limitation of 2D interfacial evaporation have allowed for developing systems with high evaporation rates. This study presents a comprehensive review of various evaporator designs that have achieved pure evaporation rates beyond 4 kg m^−2^ h^−1^, including structural and material designs allowing for rapid evaporation, passive 3D designs, and systems coupled with alternative energy sources of wind and joule heating. The operational mechanisms for each design are outlined together with discussion on the current benefits and areas for improvement. The overarching challenges encountered by SDIE concerning the feasibility of direct integration into contemporary practical settings are assessed, and issues relating to sustaining elevated evaporation rates under diverse environmental conditions are addressed.

## Graphical abstract

Recent advances in solar‐driven interfacial evaporation show promising potential for desalination and water purification. Breakthroughs have surpassed 2D limitations, achieving very high evaporation rates. This review explores innovative evaporator designs, including rapid evaporation materials, passive 3D systems, and integration with alternative energy. Operational mechanisms, benefits, and challenges for practical implementation are discussed, fueling curiosity for potential solutions.

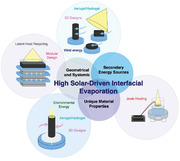

## 1 Introduction

Rapid economic growth and population expansion have increased global water supply challenges, necessitating the exploration of alternative freshwater production methods. Large‐scale desalination and wastewater treatment techniques have gained widespread adoption to meet the escalating demand for freshwater in many countries. However, these systems require substantial initial capital investments, supporting infrastructure and ongoing operational and maintenance costs. Consequently, these solutions are often unsustainable in many countries grappling with water supply issues, particularly at the community level.

Solar‐driven interfacial evaporation (SDIE) has emerged as a useful technique for freshwater generation as well as the concentration of nutrients and pollutants. Alternative terms found in literature for SDIE are solar steam generation, solar vapor generation, and solar water evaporation, all denoting the same technological concept. Historically, SDIE is based on solar still technology, wherein volumetric heating methods have been used by lining the bottom of the bulk water basin with photothermal liners.^[^ ^1^ ^]^ This traditional process has further developed into dispersing photothermal nanoparticles (NP) for a more even heat distribution in the bulk water.^[^ ^1^, ^2^ ^]^ Early volumetric configurations encountered substantial heat dissipation into the bulk water, leading to decreased evaporation rates, and convective and radiative heat loss into the atmosphere. The inefficient utilization of photothermal energy in such designs resulted to very low evaporation rates.

Consequently, research efforts have focused on strategies involving floating materials or air‐water interface systems that concentrate heat within a smaller water volume.^[^ ^3^ ^]^ These interfacial designs, referred herein as SDIE, increase evaporation rate and minimize convective heat dissipation into the larger water body. Alternative distillation modes have been introduced where the evaporation and heat transfer processes take place in separate materials and in opposite directions. This approach can recycle latent heat and has expanded the array of materials available for photothermal conversion and water conveyance. These systems tend to be more modular and emphasize practical freshwater production and collection rather than simply water evaporation.

The passive operation of SDIE is appealing for implementation in rural and less developed regions where access to energy sources from infrastructure can be limited. Solar energy systems, with minimal or no need for external energy sources, have garnered industry interest, particularly in the context of ultra‐high solar water evaporation, which has potential applications in resource recovery processes. Many current designs have limitations due to their impractical and unrefined modular design, and most mainly focus on the material performance at the air‐water interface in laboratory conditions. The theoretical evaporation rate limit for SDIE in 2D systems at 1 Sun irradiation (1000 Wm^−2^) is only 1.47 kg m^−2^ h^−1^ and so, to improve the utility of SDIE systems, higher evaporation rates with robust and long‐term sustainable operations should be addressed.^[^ ^4^ ^]^

**Figure 1:**
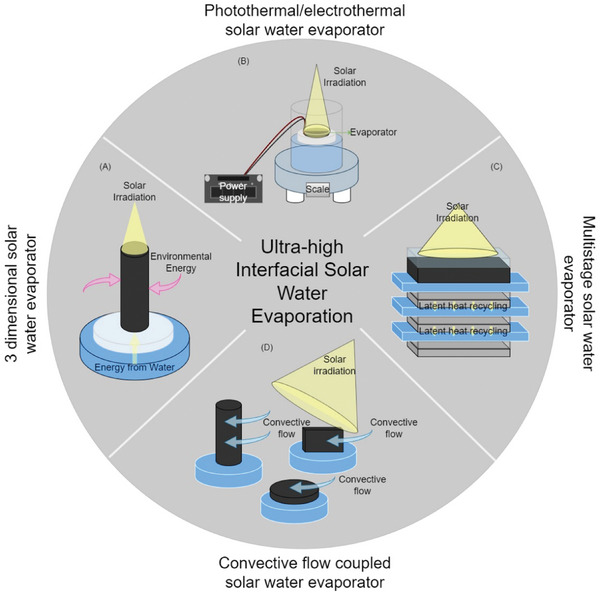
Various designs of ultra‐high solar interfacial evaporation systems: A) 3D interfacial solar water evaporator. B) Solar interfacial driven water evaporator coupled with joule heating. C) Solar driven interfacial multistage solar distiller. D) Solar interfacial driven water evaporator enhanced with convective flow.

In this review, we examine SDIE systems that have achieved ultrahigh evaporation rates above 4 kg m^−2^ h^−1^. This parameter has been chosen as SDIE mainly focuses on producing clean freshwater to address the water shortage in remote locations and population increase. It is recommended that daily fluid intake for men and women is between 2–3 L^[^ ^5^ ^]^ and ≈2 L of safe water is used per capita per day for food preparation.^[^ ^6^ ^]^ Therefore, 5 L of water per person will be needed, and for a family of 4–5, roughly 20–25 litres is required daily for consumption and food preparation. In addition, with severe water shortage issue in Africa, the South Africa Human Rights Commission has provided free basic services of 6000 L per month (25 L per day) per person to address basic rights to water sanitation.^[^ ^7^ ^]^ On average, producing 4 kg m^−2^ h^−1^ of water over an 8 h duration of sunlight will produce ≈32 L of water per day. This value has been set as a baseline for this review to facilitate consumption of average families or daily use for individuals to examine the current progress of SDIE systems available in the literature. Although practical uses of SDIE branch out to commercial and industrial applications for dewatering and various sources of wastewater treatment, the current small‐scale systems and relatively early stages of research and development present challenges to implement in those contexts. Here, we discuss SDIE with high evaporation rates based on their material composition, geometric designs, coupling with alternative energy sources and ion migration strategies (schematically presented in **Figure** **1**). We critically examine designs that generate these high evaporation rates and how they are achieved in a practical perspective.

## 2 SDIE Configurations

**Figure 2:**
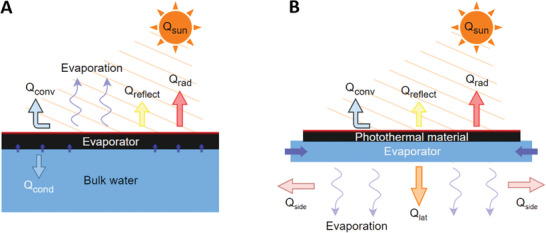
Solar interfacial evaporation design modes: A) Frontside conventional evaporation mode. B) Backside reverse evaporation mode.

The significant growth in SDIE designs spans various material compositions, modular system designs, biomimetic inspirations from plants, and integration of different technologies and techniques for desalination and water purification. SDIE systems fall into two main categories depending on the configuration of the solar absorbers, which may be in frontside conventional (FC) or backside reverse (BR) modes (**Figure** **2**). These configurations dictate the materials and functionalities essential to their SDIE performance.

The FC‐SDIE mode utilizes solar absorbing evaporators that are in direct contact with (e.g., can float on top) the bulk water. The solar absorbing evaporator is multifunctional, enabling both water flow and vapour generation within the same medium, thereby facilitating solar‐to‐thermal conversion and evaporation. The materials perform several functions: 1) converting solar energy to heat; 2) evaporation of water from the material; 3) wicking water through the material (hence requiring hydrophilicity) and; 4) thermally insulating the bulk water.^[^ ^8^ ^]^ FC‐SDIEs commonly employ carbonaceous, polymeric, semiconductor, or plasmonic materials as solar absorbers as these can be tailored to meet the required functionalities.^[^ ^8^, ^9^ ^]^ Reported FC‐SDIE modes typically do not have a condensation layer or water collection method embedded into the system; instead, they are typically placed at the top of the system, with many studies focusing on evaporation rate performance over collection rate. Hence, many studies reporting high evaporation rates may not directly translate to correspondingly high condensation or collection rates. In the FC‐SDIE mode, there is a direct correlation between the design structure of the photothermal material and the evaporation rate as the vaporization interface is at the photothermal material. Although the main effect of an increase in evaporation rate in the FC‐SDIE designs examined in this review are due to the alternative or secondary energy inputs, when examining only the photothermal (1 Sun) evaporation rate, the design structure of the photothermal layer does contribute to the overall evaporation rate. For example, a multiple light reflection 3D structure has been employed for enhanced light absorption, reduced heat loss, diffuse reflection and increased evaporation area.^[^ ^10^ ^]^

The BR‐SDIE mode involves multilayered structures (see Figure 7 as example). A typical design includes a solar absorber layer that provides thermal conversion of solar energy to heat, which can be transferred to the conduction layer. A low thermal resistance material is an important requirement as it will reduce the overall heat loss during the heat transfer process. Aluminum nitride plates have been used as thermal conduction layers due to their high thermal conductivity and resistance to corrosion in saline water.^[^ ^11^ ^]^ Transparent polyethylene‐vinyl acetate (EVA) films have also been employed.^[^ ^12^ ^]^ Heat is transferred to the evaporation layer, where an inflow of saline water occurs for desalination processes. The evaporation layer should be hydrophilic material with relatively large pore sizes to facilitate a consistent inflow of salt water.^[^ ^11^, ^12^, ^13^ ^]^ The rate of this process is critical as it can be a major bottleneck in passive multistage designs. If flow is not optimised with the evaporation rate, a “dry out” phenomenon can result.^[^ ^14^ ^]^ The hydrophilic evaporator is typically integrated with either an air gap or a hydrophobic membrane, serving as the conduit for vapor transport to a condensation layer and the collection of condensates. In the context of solar water evaporators, an air gap is the predominant choice for this purpose. Optimal thicknesses for this air gap are generally within the range of 2–5 mm to allow mass and heat transfer and prevent contamination of freshwater.^[^ ^8^, ^15^ ^]^

One can notice that passive photothermal membrane distillation functions in a similar manner to SDIE but employs a hydrophobic membrane as the interface. In this case, the hydrophobic membrane plays a critical role in this system by separating preheated feed solution from the condensed liquid.^[^ ^16^ ^]^ Due to the membrane's hydrophobic nature, liquid water cannot permeate from the evaporation layer to the condensation layer.^[^ ^17^ ^]^ Mass transfer occurs solely in the vapour phase due to the temperature‐induced partial vapour pressure differential across the membrane.^[^ ^18^ ^]^ The generated vapour passes through the hydrophobic layer onto the condensation layer where the freshwater is collected and passed out of the system. In theory, all non‐volatile solutes, including macromolecules, colloids, and ions, are subject to rejection. The membrane's low hydrostatic pressure also aids in the prevention of fouling.^[^ ^12^, ^19^ ^]^ Commonly used materials for photothermal membrane distillation processes include polyvinylidene‐fluoride (PVDF), polytetrafluoroethylene (PTFE), polypropylene (PP), and polystyrene (PS).^[^ ^11^, ^20^ ^]^

## 3 Evaporation Rate Beyond the 2D Theoretical Limit

Several recent studies have reported ultra‐high solar water evaporation rates above 4 kg m^−2 ^h^−1^, which makes SDIE attractive as a solution for freshwater production and pollutant/nutrient concentration. Many SDIE systems are passive or can utilize photovoltaic cells and so there is an appeal for industrial applications and fabrication of modular systems. Efficiencies higher than 80% are now achievable in conventional distillation modes of 3D evaporators, hydrogels, and aerogels.^[^ ^21^ ^]^ In single layer conventional distillation mode, the achievement of SDIE above theoretical limit has been actively discussed. The water evaporation efficiency can be calculated by:^[^ ^15a^ ^]^

(1) $$\eta_{w}=\frac{m_{w}h_{fg}}{AIt}$$

where *η_w_* is the water evaporation efficiency, *m_w_* is the evaporation rate (kg m^−2^ h^−1^), *h_fg_* is the latent vaporization enthalpy of liquid water (kJ kg^−1^), *A* is the area (m^2^) of solar irradiation, *I* is the incident solar irradiation (Wm^−2^) and *t* is the duration of solar illumination. The theoretical pure water evaporation limit for 2D evaporators is ≈1.47 kg m^−2^ h^−1^ assuming 100% solar‐to‐vapour energy transfer efficiency^[^ ^8^, ^22^ ^]^ at 1 Sun. Many studies have reported higher values than the theoretical limit. However, there could be some discrepancies in the calculation of evaporation rates depending on how the experimental system was designed and implemented. One recent study by Fillet et al. examined the effects of unwanted evaporation on the total evaporation rate of 2D materials.^[^ ^4b^ ^]^ Four different samples, namely: 58 mm disc (58D), 40 mm square (40S), 30 mm square (30S) and 20 mm square (20S) were tested in a 58 mm diameter beaker with changing conditions by eliminating unwanted evaporation with parafilm and limiting the aperture to the surface area of the evaporator. The 58D sample produced an evaporation rate of 1.18 kg m^−2^ h^−1^, while the 40, 30, and 20S evaporated 1.52, 2.57, and 5.07 kg m^−2^ h^−1^, respectively. When the side evaporation was restricted, the evaporation rate for the 30 and 20 S samples was reduced to 1.6 and 2.6 kg m^−2^ h^−1^, respectively. This was then further reduced to 1.11 and 1.05 kg m^−2^ h^−1^ after the aperture of the solar irradiation was limited to the sample's surface area. The evaporation rate is sensitive to the parameters set as side evaporation and increase in sunlight can induce inaccurate representation of actual evaporation of materials.

The main methods to exceed 90% of solar to vapour conversion efficiency are utilizing thermal management strategies or fabricating advanced materials.^[^ ^8^ ^]^ Ideally, increases in surface temperatures and high solar to thermal energy conversions promote increased evaporation. When looking at material‐associated evaporation rate increase, the latent vaporisation enthalpy is often decreased in some material mediums as evident in hydrogels.^[^ ^23^ ^]^ The factors influencing evaporation rates include surface area, transport pathways, heat transfer efficiency, and mass transfer dynamics. High surface area‐to‐volume ratios in both 2D and 3D structures facilitate efficient evaporation by exposing more liquid to the environment. The main difference between 2D and 3D evaporator is that the entire 2D evaporator's surface is exposed and absorbs the solar light, while 3D evaporators have additional surfaces which are not exposed to the incoming solar irradiation.^[^ ^9a^ ^]^ These additional surfaces where surface temperatures are lower than their current ambient temperature can produce higher evaporation rate utilizing other environmental energy sources such as ambient temperature and convective flow, especially in alternate geometric shapes.^[^ ^24^ ^]^ 3D structures offer enhanced transport pathways, such as capillary channels and porous networks, for efficient water supply to the evaporation surface. The intricate architecture for 3D evaporator designs should consider improved heat transfer by reducing the total heat transfer distance through the material for faster and more efficient vaporization. According to Fourier's law of heat transfer, the thermal energy flow within the evaporator is governed by its inherent thermal conductivity and temperature gradient across the heat transfer distance, as calculated by:

(2) $$Q_{\mathrm{heat}}=-kA_{\mathrm{cross}}\frac{\Delta T}{\Delta x}$$

where *Q*_heat_ is the rate of heat transfer, *k* is the thermal conductivity of the evaporator, *A*_cross_ is the cross sectional area in the direction of heat transfer, Δ*T* is the temperature difference across the evaporator, and Δ*x* is the distance over the temperature difference. Thus, evaporators with high conductivity and reduced heat transfer distance during the experimental process will display rapid heating. Additionally, the saturation of humidity near the interfaces of the evaporator, both within the medium and surface, impacts the maximum continual vaporization process. The mass transfer dynamics, including diffusion of water vapor, are optimized in 3D structures through shorter pathways and promotion of convective airflow within the material.^[^ ^9a^ ^]^ Ultra‐high solar water evaporation rates have exceeded the 2D limit from materials changing vaporization enthalpy^[^ ^25^ ^]^ and utilization of environmental energy from 3D evaporators.^[^ ^8^, ^22^ ^]^ Environmental energy in a cylindrical 3D evaporator has been estimated^[^ ^26^ ^]^ by:

(3) $$\begin{array}{ccc} E_{\mathrm{Environment}} & = & -A_{1}\varepsilon \sigma \left(T_{1}^{4}-T_{E}^{4}\right)-A_{2}\varepsilon \sigma \left(T_{2}^{4}-T_{E}^{4}\right)\\ & & -\;A_{1}h\left(T_{1}-T_{E}\right)-A_{2}h\left(T_{2}-T_{E}\right)-Q_{\mathrm{water}} \end{array}$$

where *A*_1_ is the area of top evaporator surface, *T*_1_ is steady average temperature of the top evaporation surface, A_2_ is the area of the side evaporation surface (circumferent surface), *T*_2_ is the average temperature of the circumferential surface, *T*_E_ is the ambient temperature, *ε* is emissivity of the evaporation surface, *σ* is the Stefan‐Boltzmann constant (5.67 × 10^−8^ Wm^−2^K^−4^), Q_water_ is the total energy gained from the bulk water and *h* is convection heat transfer coefficient.^[^ ^26a^ ^]^ Q_water_ can be calculated by:

(4) $$Q_{\mathrm{water}}=cM\Delta T_{\mathrm{bulk}}$$

where c is the specific heat of water, M is the total weight of bulk water and Δ*T*_bulk_ is the change of temperature of bulk water.^[^ ^26a^ ^]^

The type of material utilized by SDIE systems with high evaporation rates is critical in understanding the evaporation mechanisms that underpin their performance. Hydrogels have 3D cross‐linked structures, high hydrophilicity, and extensive networks of pore structures facilitating rapid water transport and the capacity to store substantial quantities of water.^[^ ^27^ ^]^ Hydrogels store water in three distinct states: bound water (BW), which involves water being firmly attached to the polymer; free water (FW), comprising molecule‐molecule bonding; and intermediate water (IW), characterized by weakened bonding between BW and FW.^[^ ^28^ ^]^ This unique storage state allows for water to be evaporated with less input energy than BW or FW.^[^ ^28^, ^29^ ^]^ Another hypothesis on achieving evaporation rates beyond the theoretical 2D limit has been associated with photons cleaving off water clusters.^[^ ^30^ ^]^ This unique effect has been directly associated to the photoelectric effect as tests were completed with negligible absorption of the visible spectral wavelength on the hydrogel and water. Notably, the hydrogel tested produced evaporation rates over 2 kg m^−2^ h^−1^, beyond the theoretical limit without the use of solar or light absorbers. The vapour temperature at the interface was found to be higher without light illumination, dissociating the evaporation from the photothermal effect.^[^ ^30^ ^]^ The high evaporation rate of hydrogels can possibly be associated with the photo‐molecular effect. The evaporation rate peaks are observed at the wavelength of 520 nm, corresponding to the predominance of green light, which water absorbs the least.^[^ ^30^ ^]^ However, this phenomenon remains unexplained.

Aerogels are 3D porous materials with controllable hydrophilicity and wettability.^[^ ^31^ ^]^ Similar to hydrogels, their transportation of water can be enhanced through hydrogen bonds generated through hydrophilic groups (‐OH, ‐NH_2_, ‐COOH) utilized in the material.^[^ ^31a^ ^]^ Three key elements for achieving high evaporation rates are: (1) broad spectrum wavelength absorption to enhance light‐to‐heat conversion, (2) enhanced heat management, and (3) minimization of vaporization enthalpy.^[^ ^31c^ ^]^ Charged materials and electrodes have also been explored to influence hydrogen bonds at the vaporization interface. Electronegative materials can redistribute the water molecules at the evaporator interface, weakening hydrogen bonding and enabling water to escape below its boiling point.^[^ ^28b^ ^]^ Water molecules confined within a molecular mesh tend to escape the polymer network in smaller clusters rather than as individual molecules.^[^ ^32^ ^]^ Moreover, the ultra‐high evaporation phenomenon is influenced by factors such as maintaining the saturation of the evaporator interface through rapid water transport networks.^[^ ^22^, ^27^ ^]^ This is closely linked to designs that rely on the dilution of salt concentrate to mitigate crystallization at the evaporative interface, thereby facilitating sustained high evaporation rate over extended periods.^[^ ^33^ ^]^

In the present study, we investigate methods that have achieved ultra‐high SDIE by coupling with existing and novel mechanisms. Before delving into each mechanism, it is crucial to differentiate solar‐to‐vapour conversion efficiency, freshwater collection, and pure water evaporation rates, as they have been closely linked in literature. The effects of a physical condensing cover over an evaporator have been shown to impact the ambient temperature contained within the cover, ambient relative humidity and surface temperature of the photothermal material.^[^ ^34^ ^]^ These condensation covers tend to increase the temperature for all three of the factors mentioned, but significantly increase relative humidity. The higher relative humidity prevents further increases in evaporation.^[^ ^34^, ^35^ ^]^ At lower relative humidity, a higher difference between ambient temperature and bulk water allows for more heat capture and water evaporation.^[^ ^36^ ^]^

Pure water evaporation rates and water collection from SDIE are closely related but are not the same. In many reported studies in literature, evaporation rates are usually reported but not the collection rate, wherein the latter is more important in terms of practical application. Thus, it is problematic to make direct comparisons of both in terms of performance as they ultimately serve different purposes.^[^ ^21^ ^]^ Here we mainly consider the evaporation rate as it is the most comparable value. Distillation and water collection will be examined separately where data is available to applications for freshwater collection for human use.

### 3.1 High Evaporation Rate on Frontside Conventional Modes

Designs have been implemented into frontside conventional modes to provide ultra‐high rates of evaporation. 3D evaporator designs leverage ambient energy to overcome convective heat loss. Strategies to improve evaporation rates include coupling frontside conventional evaporation modes with joule heating and convective flow. Issues of long‐term stable operation have been addressed with salt mitigation through diffusion, ion transfer and dilution strategies. One practical concern associated with SDIE is a significant reduction in water production associated with environmental factors and limitations with night‐time and low light conditions. Ambient temperature, humidity and access to solar irradiation are factors that affect the practical effectiveness of SDIE. Despite the efficiency and high performance of these materials and systems, moderating certain conditions to reach freshwater production levels suitable for daily use is a major constraint. In this section, we delve into methods that have attempted to overcome some of these environmental variables through enhancing evaporation rate by coupling both joule heating and harnessing convective flow to SDIE.

#### 3.1.1 3D Evaporator Designs

**Figure 3:**
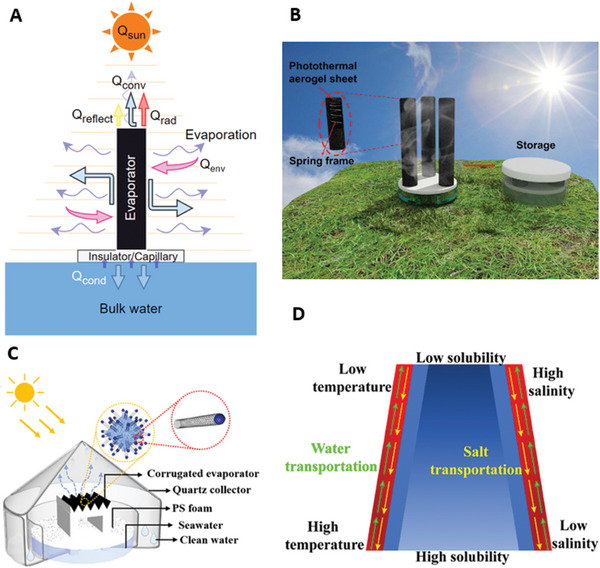
Various designs of 3D solar driven interfacial solar water evaporators: A) Schematic representing energy and mass transport in a 3D solar driven SDIE. Q_sun_ is the input solar energy converted to convective heat loss Q_conv_, radiative heat loss Q_rad_, conductive heat loss Q_cond_, reflective heat loss Q_reflect_, and environmental energy gain Q_env_. B) 3D reduced graphene oxide, sodium alginate coated cotton hollow solar driven SDIE. Reproduced with permission.^[^ ^26a^ ^]^ Copyright 2021, Wiley‐VCH; C) Schematic diagram of a 3D corrugated solar driven SDIE with Fe‐doped carbon nanotubes. Reproduced with permission.^[^ ^39^ ^]^ Copyright 2023, American Chemical Society; C) D) 3D‐printed 3D conical solar driven SDIE. Reproduced with permission.^[^ ^40^ ^]^ Copyright 2023, Elsevier.

Typically, in laboratory settings, the top surface of the 3D evaporator is directly exposed to solar irradiation, resulting in the side surfaces maintaining temperatures at or below the ambient temperature. The evaporator therefore utilizes the ambient temperature to generate cold/dark evaporation from the surrounding air.^[^ ^26^, ^37^ ^]^ **Figure** **3** shows a schematic of a generic 3D evaporator with various mass and heat flows and various 3D evaporator designs. In earlier solar interfacial water evaporator designs, 2D evaporators intrinsically have higher conductive heat loss due to a larger portion of total surface area in direct contact with (including being submerged below) the bulk water.^[^ ^38^ ^]^ This challenge has been addressed by adopting 3D indirect contact systems, whereby the photothermal evaporator is affixed to a partially submerged, thermally insulating material used for transporting water.^[^ ^26^, ^38^ ^]^ This setup increases the overall surface area exposed to solar energy and allows for taller evaporators.

As designs using 3D aerogels, hydrogels and foams become taller and thicker, some key issues arise.^[^ ^38a^ ^]^ For instance, when water saturation occurs via capillary forces throughout the entire 3D structure, vapor generation is confined to the outermost layers, while the internal volume mainly facilitates diffusion or helps maintain the structural integrity of the design. Furthermore, as these structures increase in height, the inherent fragility of the materials diminishes their ability to stand independently or imposes height limitations, thereby limiting the overall surface area.^[^ ^38a^ ^]^

##### 3D Hydrogel Evaporators

Polypyrrole (PPy) has been integrated into the structural framework of poly(N‐isopropylacrylamide) and alginate to form a hydrogel. The thermally responsive hydrogel displayed highest performance at 4 cm in height and produced deionized water evaporation rate of 4.145 kg m^−2^h^−1^ under 1 Sun irradiation. The evaporation decreased when using saltwater to 2.57 kg m^−2^h^−1^.^[^ ^41^ ^]^ No visual salt crystallization was reported and the hydrogel remained operational after being utilized seven times. The relatively significant evaporation reduction with the seawater can be explained through the increased concentration of salt and other ions at the interface from the continual inflow of seawater while water continues to evaporate.^[^ ^42^ ^]^

##### Carbon‐Based 3D Evaporators

A 3D photothermal aerogel has been fabricated by spray coating cotton sheets with a mixture of reduced graphene oxide (rGO) and sodium alginate (SA) (Figure 3B). The material was wrapped around springs for efficient transport as it can be compressed to approximately one‐third of its initial length. The 9 and 14 cm designs produced pure water evaporation rates of 6.1 and 7.6 kg m^−2^ h^−1^, respectively.^[^ ^26a^ ^]^ The total environmental energy (excluding solar irradiation) was calculated by estimating the net energy gain using Equation (2). The temperatures of the top and side surfaces of the 14 cm design during the evaporation process reached 22.4 and 15.3 °C, respectively, and did not exceed the ambient temperature of 25 °C. Consequently, there was no heat loss from the surfaces.

Alternative approaches to vertical standing geometric designs have been developed by increasing the refraction angles of solar irradiation onto the evaporator's surface. In one study, a 3D evaporator with iron‐doped carbon nanotubes (CNT) sprayed onto corrugated PVA immersed melamine foam (Figure 3C) utilized two vertical water channels transporting the bulk water into a horizontal channel, which supports the corrugated Fe/CNT surface. This fabrication process created a Janus structure, introducing a hydrophobic photothermal layer on top of the hydrophilic foam, allowing for extended operation with salt resistance and stable supply of brine. The height of the vertical water channels and surface refraction angle are optimized to 5.5 cm and 60°, respectively. This design demonstrated continuous operation over a 12 h period, with diminishing performance down from 4.2 kg m^−2^ h^−1^ at salinity of 3.5 wt.% to 3 kg m^−2^ h^−1^ at 21 wt.%.^[^ ^39^ ^]^ Although, the performance diminished below ultra‐high evaporation rates with higher salt concentration, it is still competitive with other high performing systems.

##### Geometrical Manipulation of 3D Evaporators

3D‐printed solar interfacial evaporators with volcano‐like structures have been fabricated using fused deposition modelling (FDM) (Figure 3D). Construction utilized a black polylactic acid (PLA) substrate and subsequently coated with polydopamine (PDA) to enhance its hydrophilic characteristics.^[^ ^40^ ^]^ PLA by nature is not a hydrophilic material, therefore tests were conducted on different PDA dosages to impart hydrophilic properties, whereby rapid water transport is possible. Presently, PLA is the predominant material of choice for 3D printing due to its affordability, eco‐friendliness and ease in processing.^[^ ^40^, ^43^ ^]^ Consequently, strategies aimed at enhancing hydrophilicity in such materials are important for 3D‐printed solar water evaporators and manufacturing processes. The volcano‐like configuration, with a height‐to‐diameter ratio of 1 (15 mm), maximized the potential of the Marangoni effect from the temperature gradient for upward water transportation. Coupled with three layers of PDA, this design yielded an evaporation rate of 4.02 kg m^−2^ h^−1^.^[^ ^40^, ^44^ ^]^

Solar‐driven interfacial 3D evaporators have displayed stable and high evaporation rates with the use of solar and passive environmental energy. They provide applications to water production as well as resource recovery from seawater, urine, and wastewater.^[^ ^45^ ^]^ An example of this is the extraction of lithium from saline water through ultrahigh evaporation to induce salt crystallization on a cellulose fiber string. The cellulose fiber crystallizing string was fabricated by spinning the cellulose fiber into thin string and four strings were twisted together to make 70 cm long strands. These strings are able to draw up saline water and generate high evaporation rates of up to 3.7 and 9.8 kg m^−2^ h^−1^ under outdoor conditions with 1.5 ms^−1^ of wind and 500 Wm^−2^ of solar irradiation in laboratory conditions, respectively.^[^ ^46^ ^]^ By maximizing the use of this high evaporation rate, the cellulose strings were able to generate both NaCl and LiCl crystals along the outer surfaces. As the salts with higher concentrations crystalize first, less concentrated salts crystallize further up on the fiber and further outside of the crystallized shell. This physically separates the salt crystals and allows for ease in extraction. These kinds of designs can be utilized to produce other resources such as cobalt, nickel and uranium from seawater and brines.^[^ ^46^ ^]^

#### 3.1.2 Coupling Joule Heating with SDIE

**Figure 4:**
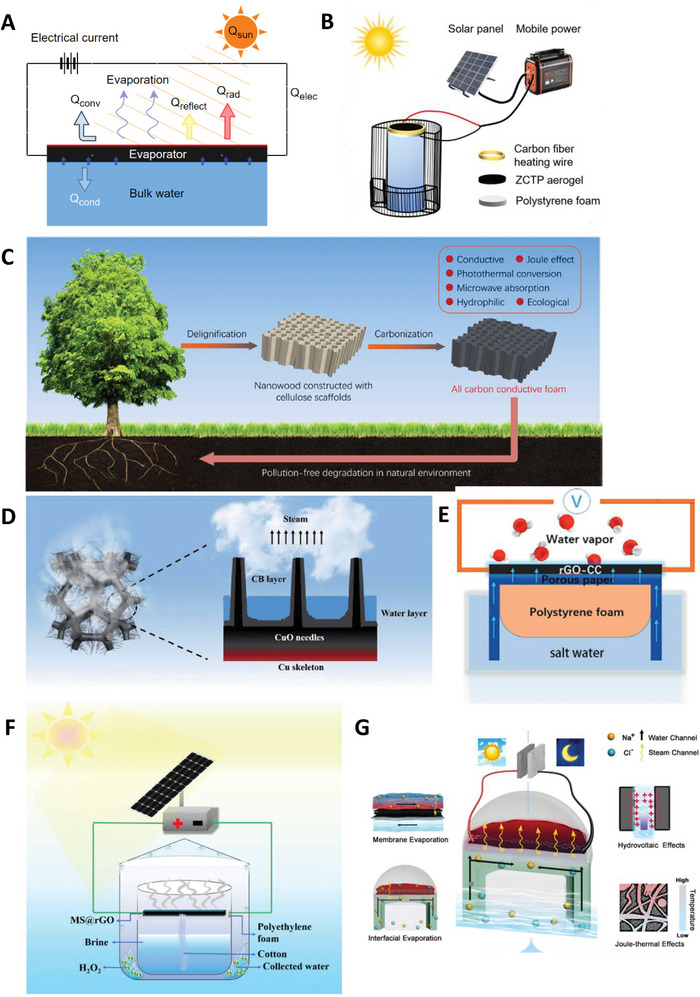
Various designs of SDIE coupled with joule heating: A) Schematic representing energy and mass transport in a SDIE coupled with joule heating. Q_sun_ is the input solar energy converted to convective heat loss Q_conv_, radiative heat loss Q_rad_, conductive heat loss Q_cond_, reflective heat loss Q_reflect_. B) All weather SDIE 3D zeolite‐chitosan‐TiO_2_@PPy aerogel. Reproduced with permission.^[^ ^50^ ^]^ Copyright 2023, Elsevier; C) All weather natural wood derived carbon conductive foam. Reproduced with permission.^[^ ^52^ ^]^ Copyright 2023, Elsevier;. D) Schematic of CuO/Cu–CB foam. Reproduced with permission.^[^ ^53^ ^]^ Copyright 2023, Elsevier; E) Low voltage graphene carbon cloth (CC)‐based carbon‐carbon composites. Reproduced with permission.^[^ ^54^ ^]^ Copyright 2020, Wiley‐VCH. F) Photothermal and electrothermal melamine sponge (MS)‐supported reduced graphene oxide. Reproduced with permission.^[^ ^55^ ^]^ Copyright 2022, Wiley‐VCH; G) Schematic diagram of monolithic all‐weather solar thermal interfacial solar membrane evaporator. Reproduced with permission.^[^ ^56^ ^]^ Copyright 2022, Elsevier.

**Figure** **4** presents some new SDIE designs utilising Joule heating as an additional evaporation energy source. Joule heating is used to increase the temperature of a liquid through the resistance of an electrical current.^[^ ^47^ ^]^ This allows the water production rate to be controlled during low sunlight periods and generate stable evaporation during night‐time operation. These materials and systems are most often used in frontside conventional distillation modes, where the photothermal evaporator material are electrodes that generate electrical resistance. This resistance then produces heat within the medium, increasing temperature and thus evaporation from the bulk water. This mechanism is not passive and requires an external power source usually 2–5 V. PV cells for enhanced Joule heating processes have displayed a great potential in combination with photothermal energy.^[^ ^48^ ^]^ Other methods for generating electricity have been through hydrovoltaic effect, pyroelectric effect and nanogenerators.^[^ ^49^ ^]^ Figure 4A shows mass and energy flows in this set‐up.

##### Zeolite Aerogel Evaporator for Joule Heating

A photothermal‐electrothermal integrated system using zeolite‐chitosan TiO_2_@PPy aerogel (ZCTP) has been coupled with PV cells in an out‐door environment to self‐sustain energy input, Figure 4B.^[^ ^50^ ^]^ Prior to implementing the electrothermal energy, the evaporator generated 1.66 kg m^−2^ h^−1^ under 1 Sun. Applying 5 V voltage under 1 Sun achieved an ultra‐high water evaporation rate of 11.7 kg m^−2^ h^−1^.^[^ ^50^ ^]^ When tested in outdoor environments, the evaporator produced 6.82–8.2 kg m^−2^ over a 10 h period during the day (9:00–19:00) and 4.02–5.02 kg m^−2^ over a 10 h period during night‐time (19:00–9:00). In addition, although, the water production rates are below 1 kg m^−2^ h^−1^, it displays the potential for all day freshwater production regardless of the time and weather conditions.

##### Carbon‐based Materials for Joule Heating

Carbon‐based materials possess the critical elements for photothermal and electrothermal processes. They are more accessible and lower in cost compared to metallic materials and semiconductors with a broad light absorption spectrum, thermally conductive properties, high efficiency in solar to heat conversion efficiency and are electrically conductive.^[^ ^9^, ^51^ ^]^

All‐carbon conductive foam (ACCF) derived from natural wood has been proposed for all‐weather evaporation applications (Figure 4C).^[^ ^52^ ^]^ The choice of natural wood in distillation systems arises from its intricate porous structure, which enhances hydrophilic properties and salt rejection capabilities. Lignin and hemicellulose were eliminated from wood and subjected to carbonization in an argon atmosphere at various temperatures: 1000, 1200, and 1400 °C (C‐1000, C‐1200, and C‐1400) to enhance its solar absorption capabilities. The C‐1200 natural wood‐based evaporator achieved the highest freshwater production rate of 2.25 kg m^−2^ h^−1^ under 1 sun illumination and 6.73 kg m^−2^ h^−1^ when supplied with 2 V for electrothermal heat generation.^[^ ^52^ ^]^ In another study, an all‐day vapour generator was fabricated by chemically etching a copper oxide (CuO) needle array on the surface of a copper foam substrate (Figure 4D). The photothermal aspect of the material was developed by depositing a layer of carbon black (CB) nanoparticles onto the CuO/Cu foam. The CuO/Cu‐CB achieved a pure water evaporation rate of 1.65 and 4.5 kg m^−2^ h^−1^ under 1 Sun and 2 V of input energy, respectively.^[^ ^53^ ^]^ Figure 4E shows flexible rGO mixed carbon cloth prepared using an electrochemical process. The material was able to generate a steady state temperature of 389 °C with an input voltage of 3.5 V displaying its high electrothermal properties. For pure water evaporation rate, evaporator managed to reach 2.54 kg m^−2^ h^−1^ (at 1 Sun) and when supplied with 3 V, the evaporation rate increased up to 26.52 kg m^−2^ h^−1^. Notably, the hydrophobic carbon material prepared through an electrodeposition method managed to reach 2.4 and 26.52 kg m^−2^ h^−1^ by inputting 1 Sun irradiation and 3 V, respectively.^[^ ^54^ ^]^

Increasing the voltage to these electrothermal systems results in greater heat generation, which, in turn, enhances the rate of water evaporation. However, at high voltage, electrolysis may occur at the water surface, leading to the production of hydrogen and oxygen gases instead of the intended vaporization of water.^[^ ^56^ ^]^ This unintended electrolysis reduces the overall efficiency of the system and there is an elevated risk of material degradation occurring at the interface of the evaporator. Furthermore, excessive voltage levels may induce boiling at the water interface, leading to increased heat loss, scaling, and fouling at the evaporator interface. These issues can significantly reduce the economic feasibility of the process due to heightened energy consumption.

Evaporators have been constructed using affordable commercial melamine sponge and incorporated well‐dispersed graphene oxide (GO) to evenly wrap the sponge substrate framework (Figure 4F).^[^ ^55^ ^]^ The GO layer on the framework was subsequently reduced using hydrogen iodide (HI) to prevent the reaggregation of the reduced GO. This resulted in the creation of the MS@rGO (melamine sponge wrapped with reduced graphene oxide), which exhibited conductive properties suitable for electrothermal processes, combined with photothermal heating, to achieve ultra‐high evaporation rate. The evaporation achieved 1.9 kg m^−2^ h^−1^ under 1 Sun, and 9.02 kg m^−2^ h^−1^ under combined 1 Sun and 10 V of electricity.^[^ ^55^ ^]^ Applying 5 V of electricity would result in an evaporation rate of ≈3–3.5 kg m^−2^ h^−1^. The effect of salinity on the rate of evaporation relies on a specific concentration of salt that is necessary to facilitate the transfer of electrons within the bulk water. Additionally, the collision of surface photogenerated electrons contributes to an increase in heat within the evaporator, ultimately leading to an ultra‐high evaporation rate.

An all‐weather solar‐thermal interface evaporation system (STIME) (Figure 4G) utilized a hybrid system using MXene‐graphene oxide‐MXene (MGM) on a commercial PVDF membrane, capable of joule‐heating for water evaporation.^[^ ^57^ ^]^ The STIME produced 1.18 kg m^−2^ h^−1^ under 1 Sun and has the capability to produce 10.5 kg m^−2^ h^−1^ under 0.5 Sun and 36 V. Notably, the total electricity input in this system surpassed that of many other research outputs. Generally, the evaporator medium often struggles to withstand such high voltage levels, leading to structural breakdown. This research primarily focuses on high durability characteristics and explored possibilities in various nutrient recovery processes rather than freshwater production efficiency, given the usage of exceptionally high voltage. To enhance hydrophobicity and reduce electric energy loss related to hydrolysis, the PVDF membrane underwent treatment with stearic acid.^[^ ^57^ ^]^

Coupling SDIEs with joule heating offers advantages in stabilizing the evaporation rate across diverse environmental conditions. SDIE systems integrated with joule heating typically attain elevated surface temperatures due to the input of two energy sources into the medium. Significant performance disparities exist between solely harnessing solar energy input and coupling with electrothermal process, the rapid evaporation and high surface temperature can result in a dry out phenomena and salt crystallization issues which needs to be considered.^[^ ^58^ ^]^ Diffusion and dilution through rapid water transport has been the main method to manage the salt accumulation the high evaporation designs evaluated above.^[^ ^52^, ^53^, ^55^ ^]^ Wang et al. tested the salt rejection through a constant extended operation of 8 h and a salt reflux test where the deposited salt was fully dissolved in 120 min for the C‐1200 design.^[^ ^52^ ^]^ Similarly, Guo et al. deposited 2.5 g of sodium chloride crystals on the surface and was dissolved within 15 min.^[^ ^53^ ^]^ Zhang et al. treated the bottom surface of the MGM@PVDF membrane with stearic acid for a hydrophobic treatment, allowing for a separation barrier between the salt water and vapor.^[^ ^57^ ^]^

The inability of solar driven evaporation processes to operate during night‐time has significantly limited the water evaporation and freshwater production to roughly 8 h per day.^[^ ^59^ ^]^ The electrothermal process serves its advantages from a practical perspective as it is more reliable and consistent, displaying clear improvements in evaporation rates. The main downside of this technology lies in the need for external energy input, especially in systems requiring higher voltage inputs. As mentioned above, water splitting can result from higher voltages above 2–3 V where efficiency is substantially reduced and can impose degradation in materials used. Minimizing voltage input while maintaining high evaporation can be suggested for long term benefits such as stability and reliability.

#### 3.1.3 Photothermal Evaporation Coupled with Convection Flow

**Figure 5:**
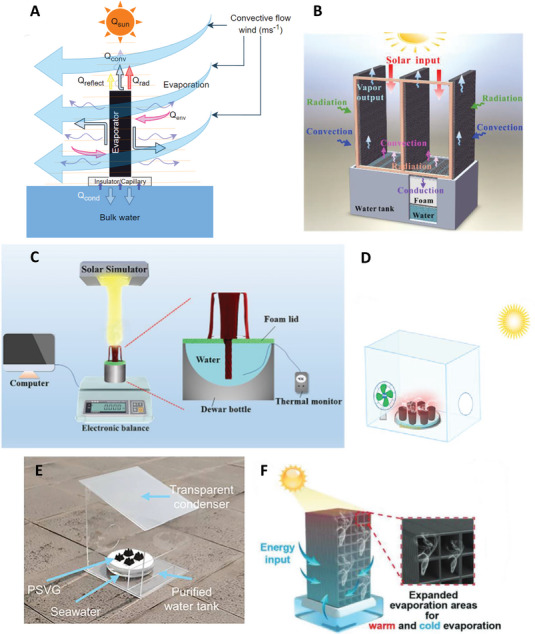
Various designs of SDIE enhanced with convective flow: A) Schematic representing energy and mass transport in a SDIE supported with convective flow. Q_sun_ is the input solar energy converted to convective heat loss Q_conv_, radiative heat loss Q_rad_, conductive heat loss Q_cond_, reflective heat loss Q_reflect_, environmental energy gain Q_env_. B) 3D solar water evaporator with multi‐vertical sheets. Reproduced with permission.^[^ ^60b^ ^]^ Copyright 2022, Elsevier; C) 3D opened hollow photothermal evaporator. Reproduced with permission.^[^ ^37c^ ^]^ Copyright 2022, Wiley‐VCH; D) 3D solar driven super‐hydrophilic aerogel with rGO and PVAP. Reproduced with permission.^[^ ^27b^ ^]^ Copyright 2023, Elsevier. E) 3D printed electro spun nanofiber‐based pyramid‐shaped solar vapor generator. Reproduced with permission.^[^ ^60a^ ^]^ Copyright 2023, Elsevier; F) 3D printed 3D self‐standing aerogel matrices. Reproduced with permission.^[^ ^38a^ ^]^ Copyright 2023, Wiley‐VCH.

Convection flow coupled with photothermal evaporation (**Figure** **5**A) is another emerging method to generate ultra‐high evaporation rates. These evaporators have reached evaporation rates from 10 to almost 30 kg m^−^^2^h^−1^.^[^ ^22^, ^27^, ^37^, ^60^ ^]^ Liquid transfer processes in evaporation systems can be accelerated utilising convective flow by applying wind between 2–6 ms^−1^ on the evaporation surface. These speeds are categorized as a light‐ to moderate breeze according to the Beaufort Wind Scale^[^ ^61^ ^]^ and is often accessible in urban regions and readily accessible in the proximity of coastal areas. The movement of air through the evaporation interface allows for a reduction in vapour density, resulting in a vapour diffusion process to maintain the equilibrium. This, in turn, results in a drastic increase in the evaporation rate. Many of these evaporators have been tested in open, outdoor environments and maintained ultra‐high evaporation rates while displaying the benefits of utilizing solar energy. To facilitate convective flow, it is essential for the wind to directly interact with the evaporation surface. However, the inherent nature of this process means that when a vapor‐collecting condenser system covers the setup, it can impede or entirely block the access of wind. Therefore, as discussed above, there is a clear need to distinguish the evaporation rate and the water collection rate.^[^ ^21^ ^]^

Many convection flow systems coupled with photothermal energy considerably outperform other designs in terms of evaporation rate. However, these designs require wind speeds of more than 4 ms^−1^ to maximize evaporation rate. On the Beaufort Wind Scale, wind speeds between 3.4–5.4 and 5.5–7.9 ms^−1^ are regarded as gentle breeze and moderate breeze, respectively.^[^ ^61^ ^]^ Consistent access to these wind speeds is primarily determined by the geographical location. However, a condenser employed for freshwater collection can hinder access to convective flow. Consequently, practical applications of these designs, especially those necessitating wind speeds greater than 3.4 ms^−1^, may be unviable in urban settings. Considering this, obtaining these wind speeds could be challenging, leading to reduced practical feasibility of the aforementioned designs. Nevertheless, studies have explored the development of evaporators that function effectively with lighter wind conditions between 2–4 ms^−1^, making them more readily available regardless of the geographical location.

##### Fabric 3D Evaporators for Convective Flow

A 3D vertical evaporator was fabricated using commercially available black cloth composed of pure cotton and a 3D printed nylon (PA200) frame (Figure 5B).^[^ ^60b^ ^]^ The black fabric was arranged in a vertical orientation with uniform gaps between each piece. Performance was assessed using various quantities of fabric (ranging from 1 to 6 pieces) and different spacings between them (ranging from 4 to 20 mm). Two designs stood out in the investigation being 3 cloth layers with 8 mm spacing (3S) and 6 cloth layers with 4 mm spacing (6S). The 3S design managed to achieve 3.32 kg m^−2^ h^−1^ under 1 Sun irradiation and the 6S reached an evaporation rate of 28.4 kg m^−2^ h^−1^ under 1 Sun and 6 ms^−1^ of convective flow.^[^ ^60b^ ^]^ The design evaporated 70.7 kg m^−2^ over an 8 h period in an outdoor open experiment.

In another study, a 3D opened hollow photothermal evaporator was fabricated using a CuS‐cellulose composite (CSC) spray coated cloth (Figure 5C).^[^ ^37c^ ^]^ The cloth coated with CSC was draped over the steel frame in such a manner that each protruding strand had a specific gap between them, allowing the evaporating moisture inside the hollow frame to dissipate. These specific slots were 0.3, 1.28, 2.19 cm, and impacted the photothermal evaporation rate by producing 3.939, 3.461, 3.024 kg m^−2^ h^−1^ under 1 Sun, respectively. Using the design with the smallest slot, the system reached 9.044 kg m^−2^ h^−1^ under 1 Sun and 2 ms^−1^ of convective flow, and 11.911 kg m^−2^ h^−1^ under 1 Sun and 4 ms^−1^ of convective flow.

##### 3D Aerogel with Convective Flow

A 3D hybrid aerogel constructed via a hydrothermal process employed rGO and polyvinyl alcohol phosphate ester (PVAP) as components to create a solar‐absorbing evaporator (Figure 5D). Different heights of the material were tested and the 3 cm tall rGO/PVAP (rGP) was determined to have the best performance of pure water evaporation rate of 4.89 kg m^−2 ^h^−1^ under 1 Sun, and 16 kg m^−2^ h^−1^ under combined 1 Sun and 2.5 ms^−1^ over 7 days. From a more practical perspective, the system underwent testing using brine with concentration of 3.5 wt.% over 8 h under 1 Sun and 2.5 ms^−1^ of convection flow, producing 14.53–15.31 kg m^−2^ h^−1^ with no salt accumulation.^[^ ^27b^ ^]^ An interesting observation during outdoor testing was the ability of sidewalls to absorb solar energy. Unlike indoor testing setups where only the top surface of the materials typically receives solar irradiation, this outdoor setup demonstrated the enhanced performance of 3D evaporators compared to 2D ones. This is due to the changing angle and intensity of sunlight throughout the day, which directly affects the total solar energy absorbed by the photothermal material. Outdoor testing of the evaporator was conducted both with and without a condenser. In the case of the system with a condenser, it operated as a closed system and did not make use of convective flow. Without a condenser, the cumulative evaporation reached 13.35 kg m^−2^ h^−1^ and 97.88 kg m^−2^, while with a condenser, the evaporation rate was 7.48 kg m^−2^ h^−1^ and 49.45 kg m^−2^.^[^ ^27b^ ^]^

The drawbacks commonly found in most 3D aerogels and hydrogels have been critiqued^[^ ^38a^ ^]^ and many materials have a significant portion of their internal pore channels unexposed as an evaporative layer and act primarily for diffusion and structural support.

##### 3D Printing for Convective Flow

In response to the drawbacks of conventional 3D hydrogels and aerogels, a 3D ink‐extrusion printing technique was used to create a self‐supporting 3D structured aerogel framework (Figure 5F). This innovative approach allows the internal pores of the structure to be accessible and utilized as an effective surface for both convective and evaporative processes. The ink was made of bacterial cellulose (BC, 2.5 wt.%), sodium alginate (SA, 0.2 wt.%), glutaraldehyde (GA, 20 mg g^‐1^) as cross‐linking agent, and carbon nanopowder as the photothermal material.^[^ ^38a^ ^]^ Four different lattice structures and 2 different heights were constructed. The design with 4.5 × 4.5 mm^2^ pores in the lattice structure with a height of 4 cm, produced the highest evaporation rate of 3.74 kg m^−2^ h^−1^ under 1 Sun irradiation. The same design also had the highest evaporation of 25.3 kg m^−2^ h^−1^ under 1 Sun, coupled with 2 ms^−1^ of convective flow. An outdoor experimental set‐up involved an all‐in‐one system where a solar‐powered ventilator for an induced airflow path was set up in a condensation chamber contained within a larger transparent evaporation chamber. This process allows for the water to be collected while taking advantage of the convective flow to reach the large surface area of the 3D aerogel. However, there was a significant difference in the performance with and without the airflow as the collection rate reduced from 18–30 to 11–17 kg m^−2^ day^−1^.^[^ ^38a^ ^]^

A 3D‐printed pyramid‐shaped electrospun nanofiber solar water evaporator was fabricated using a homogenized electrospun PAN/CNT nanofiber membrane.^[^ ^60a^ ^]^ The model with a height of 2 cm was mainly used for the experimental process and produced 2.62 kg m^−2^ h^−1^ under 1 Sun and 8.31 kg m^−2^ h^−1^ when coupled with a convective flow of 4 ms^−1^. In outdoor conditions, the material was covered by a transparent condenser with small holes in the sidewall for convection flow to occur. After 8 h of operation, the cumulative evaporation rate reached 20.1 kg m^−1^. However, it is not explicitly mentioned if this value is directly associated with the evaporated water collected.

Evaporators used in conjunction with convective flow for both pure evaporation and processing brine water have demonstrated their capacity to produce significant amounts of freshwater. However, the implementation of freshwater collection (which may block airflow) devices with these systems remains a challenge. As discussed above, various designs of condensers have been tested and have substantially reduced the evaporation rate to the extent where other 3D designs have outperformed. In future research, it may be advantageous for convective flow‐induced designs to divert some focus toward the concentration of pollutants and resource recovery as there are many industries, such as wastewater, textiles and manufacturing, where there is a demand for energy‐efficient liquid evaporation processes.

#### 3.1.4 Coupling of Ion Transfer with SDIE

Ion transfer and small‐scale electrodialysis in modular solar water evaporation systems do not directly increase the solar water evaporation rate. These methods are currently used as electrochemical separation techniques with great potential for producing freshwater and resource recovery from brine.^[^ ^62^ ^]^ Electrodes and implementation of electric potential to form a charge difference in multilayered hydrogels and membranes have displayed potential in decreasing the need for maintenance. As the cations and anions can be separated the evaporator prevents salt crystallization, and potentially lowering scaling.

The traditional passive methods of preventing salt saturation and crystallization on the evaporation surface have relied on brine diffusion and salt saturation dissolution.^[^ ^64^ ^]^ When salt crystallization occurs on the interface, it obstructs the vapour transportation due to the crystals, leading to a significantly reduced evaporation rate.

**Figure 6:**
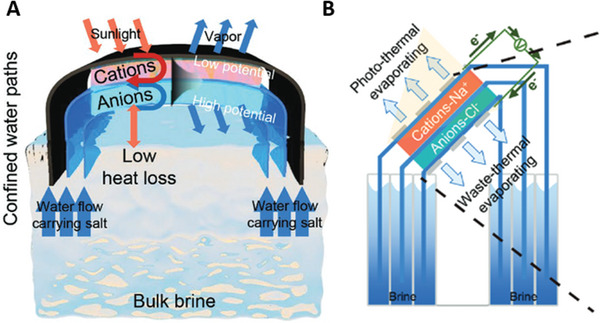
Schematic of Janus ion‐selective hydrogel for long term operation and salt mitigation. Reproduced with permission.^[^ ^63^ ^]^ Copyright 2023, Wiley‐VCH.

An ion‐selective Janus hydrogel was developed utilising a stacked cationic and an anionic hydrogel to initiate ion‐electromigration to prevent salt build‐up.^[^ ^63^ ^]^ Two separate water channels were lined on the top and bottom of the cationic and anionic hydrogel for bulk water transportation and one in between to remove the saturated brine removal. In this process, the disparity in charge within the hydrogels facilitates the downward migration of cations and the upward movement of anions toward the central removal channel to mitigate salt saturation.^[^ ^65^ ^]^ This system was operated for 7 days with 15 wt.% brine and achieved an evaporation rate of 6.86 kg m^−2^ h^−1^ under 1 Sun irradiation.^[^ ^63^ ^]^ In outdoor testing, the system achieved an evaporation rate of 4.23 kg m^−2^ h^−1^ under an average of 0.8 solar irradiation between 11:00 and 14:00. This system sustained long‐term operation and ultra‐high evaporation rates with high brine‐saturated bulk water through evaporating on both frontside and backside directions by manipulating the water transport mechanism. As seen in **Figure** **6**, three distinct setups were used, each yielding evaporation rates of the thermal waste surface generating more than 1 kg m^−2^ h^−1^, surpassing the typical frontside evaporation approach. This design exhibited encouraging outcomes in treating high salinity brine, showcasing long‐term stability and exceptional performance in real‐world scenarios. The effective implementation of a freshwater collection method could substantially enhance the practical utility of this system, particularly considering its remarkable evaporation rate.

### 3.2 High Evaporation Rate on Backside Reverse Distillation

**Figure 7:**
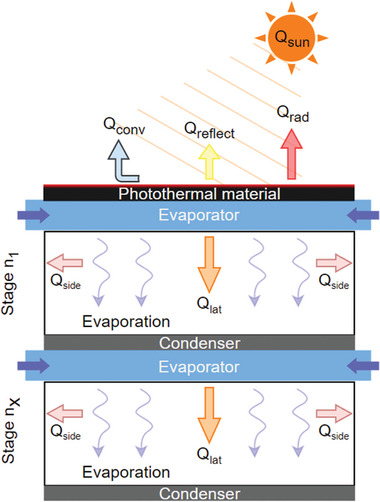
Schematic representing energy and mass transport in a multistage solar driven ISWE. Q_sun_ is the input solar energy converted to convective heat loss Q_conv_, radiative heat loss Q_rad_, conductive heat loss Q_cond_, reflective heat loss Q_reflect_, environmental energy gain Q_env_, latent heat recycles Q_lat_.

Recently, there have been developments in reverse distillation techniques which separate the solar absorber from the evaporator. This expands the range of available materials and changes how the evaporation system functions. The main advantage of this approach lies in the ability to individually optimize and choose materials for each different layer, as illustrated in **Figure** **7**. The separation of solar absorber, evaporation layer, and condensation layer facilitates the reverse distillation process. The thermal conductivity and performance of the absorber are critical as, unlike conventional front‐side evaporation, there is a process of conductive heat transfer between the evaporator and the backside photothermal absorber. During this process, evaporators/capillary wick materials are commonly constructed of low‐cost commercially available such as cellulose fiber, cotton fiber and hydrophilic polymer fiber materials. As these materials generally have lower thermal conductivity, the total heat production and heat transfer through the solar absorber need to be highly efficient. While maintaining low cost and accessibility to materials possesses its significance in backside reverse distillation, the main material used for the photothermal layer is a spectrally selective absorber, usually in a form of spray coating a metal plate (aluminum, copper) with black paint, commercially available solar absorbers and use of polycrystalline and monocrystalline photovoltaic (PV) cells.^[^ ^66^ ^]^ Segregation of functionality allows for the materials to be obtainable from cost‐effective, commercially available products, avoiding the complexities of conventional evaporator manufacturing processes. However, when directly comparing a backside reverse distillation system to a traditional or conventional frontside distillation setup, the evaporation rate and solar‐to‐vapour conversion efficiencies are typically lower due to the heat loss from conduction and sidewall convection between the layers. Typically, when evaluating reverse distillation methods, it is more relevant to consider condensation and water collection rates as a condensation layer is most often included in the set up.

#### 3.2.1 Multistage Backside Reverse Distillation

Backside reverse distillation modes require conductive and convective heat transfer down from the photothermal absorber. This process allows some residual heat to remain after condensation occurs. When utilizing thermally conductive materials for the condensation layer, the latent heat remaining in the system can be recycled in another proceeding stage and has been referred to as a multistage reverse distillation mode. Multistage reverse distillation mode mainly involves the layers outlined in the first stage in Figure 7, followed by a repeating 3‐layer stage consisting of an evaporator, hydrophobic membrane or air gap layer, and a condensation layer. These three layers can be repeated until the remaining latent heat in the final condensation layer surpasses the ambient temperature, as condensation occurs when the vapour temperature within the system remains above the outside temperature.^[^ ^8^ ^]^ It is evident in literature that the use of dense, thermally conductive metals is attractive and have been sufficient in providing enough heat for multistage configurations.

Economic and environmental factors have an influence on the number of stages as more materials are needed, and the system becomes more complex for long term maintenance and use. Another factor to consider is the need for sidewall insulation. Unlike other FC‐SDIE systems discussed above, multistage BR distillation modes include a condensation process into latent heat recycling within the module. This directly associates with the amount of distillate produced and highlights the need to maximize heat retention within the device. Thus, making it more sensitive to the total heat loss and a main parameter to consider for optimization. To reduce heat loss to the surrounding environment, the sidewall of the multistage condenser system may be sealed with a material with low thermal conductivity. For example, lining the side walls with polyurethane (PU) foam, polyethylene (PE) foam and linear low‐density polyethylene (LLDPE) to minimize convective heat loss into the atmosphere.^[^ ^11^, ^13^, ^67^ ^]^

**Figure 8:**
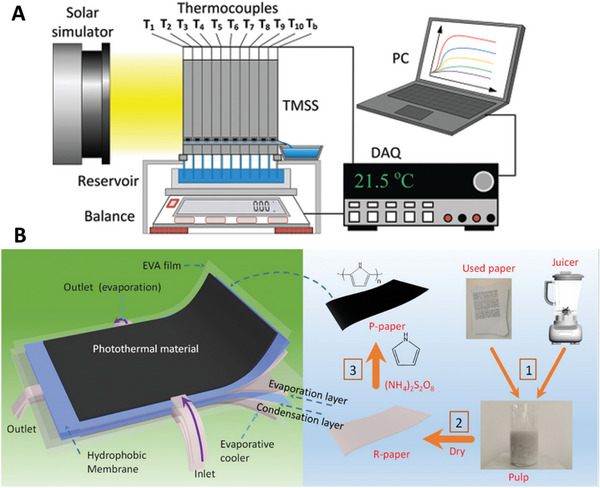
Various designs of solar‐driven multistage interfacial solar distillers: A) Schematic diagram of set up for thermally localized multistage solar‐still. Reproduced with permission.^[^ ^15c^ ^]^ Copyright, Springer; B) Passive, flexible multistage membrane distillation. Reproduced with permission.^[^ ^68^ ^]^ Copyright 2021, American Chemical Society.

**Figure** **8**A shows a design that achieved a 5.78 kg m^−2^ h^−1^ evaporation rate of pure water under 1 Sun irradiation.^[^ ^69^ ^]^ The module was a 10‐stage thermally localized multistage solar still with a 3D printed framework and passive water flow system.^[^ ^69^ ^]^ The design experienced salt crystallization around its corners after 2 h of operation when tested with 3.5 wt.% of saline water and ≈45% of the evaporative surface area after 3.5 h of operation. The device operated under a dead‐end design, relying on gravity and capillary forces for transportation of water across the evaporation area.

Due to the complexity of multistage distillation modes, salt removal and water transportation within the systems are difficult to manage and maintain over long periods of time. Crossflow modes have offered a partial solution by ensuring a consistent, steady flow of incoming feed water, either through gravity or mechanical pumping. In this approach, the saturated brine continuously exits the system before salt crystallization or oversaturation contaminants can build up. When managed well, crossflow designs can maintain long‐term stable operation without the need for frequent cleaning or replacement of layers in the multistage system.

A passive 8‐stage flexible membrane distillation system evaporated and collected 5.15 kg m^−2^ h^−1^ under 1 Sun irradiation from a pure water source (Figure 8B).^[^ ^68^ ^]^ The system utilized a solar absorber fabricated from recycled paper pulp, treated with a polypyrrole solution for the photothermal layer. The evaporation layer was also synthesized with the same pulp. When inputting a real seawater source pre‐treated with 10 ppm of nitrilotriacetic acid (NTA), the dead‐end design displayed a clean water production rate of 4.34 kg m^−2^ h^−1^ and reduced to 1.39 kg m^−2^ h^−1^ over an 8 h duration under 1 Sun irradiation. The significant reduction in production rate was due to the build‐up of salt in the evaporation layer.^[^ ^68^ ^]^ A passive siphon flow‐induced crossflow design under the same conditions produced an average of 3.61 kg m^−2^ h^−1^.

Unlike traditional or conventional frontside distillation modes, multistage reverse distillation requires more material relative to the number of stages and material composition of each layer. In addition to this, there is limited cost analysis factoring in maintenance rate/cost, lifespan, payback period, salvage values and interest rate in the literature.^[^ ^66^, ^70^ ^]^ Improvements to mass transfer in the different layers would improve multistage systems, which could be achieved mechanically and by manipulating the materials. Brine water transfer, especially through a passive method, requires initial saturation time. According to the material, the duration of total saturation time fluctuates, where the least amount of time will promote an increased evaporation area in a shorter duration, promoting an enhanced evaporation rate.^[^ ^71^ ^]^ Fan‐forced airflow in the vapour chamber allows for water molecules to transport onto the condensation surface faster, thereby improving the water collection rate.^[^ ^70^ ^]^ To facilitate the collection of water, applying a hydrophobic coating to the condensation layer enhances the rate at which freshwater is collected in systems that rely on gravity. For example, coating the condenser with a hydrophobic Teflon AF layer optimized dropwise condensation in the associated device.^[^ ^69^ ^]^

From a practical perspective, a key component in solar driven multistage distillation systems is the number of stages in its design. The number of stages ultimately determine performance and the cost of the system as performance improvement significantly decreases after 15 stages.^[^ ^8^ ^]^ Solar driven multistage systems are modular devices with water collection already implemented into their operating mechanism. The multilayered structure and each layer's reliance on one another results in the design being more complex for salt management systems. Due to this, the systems have a more complete modular design compared to frontside conventional modes where evaporation tends to be the focus of research. Its strengths lie in the distiller's ability to be employed practically if performance in real‐life environments for water production, water quality and stability can justify the device's cost and feasibility.

#### 3.2.2 Co‐Production of Water and Electricity for Self‐Reliance

**Figure 9:**
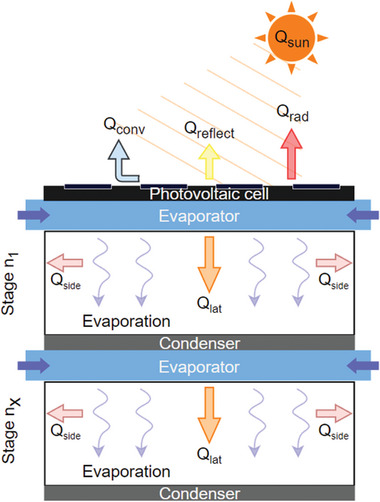
Schematic representing energy and mass transport in a photovoltaic cell multistage solar driven SDIE. Q_sun_ is the input solar energy converted to convective heat loss Q_conv_, radiative heat loss Q_rad_, conductive heat loss Q_cond_, reflective heat loss Q_reflect_, environmental energy gain Q_env_, latent heat recycles Q_lat_.

Low levels of electricity input are necessary to amplify the evaporation of photothermal evaporators with electrothermal processes. Systems needing low voltage joule heating mechanisms may be difficult to implement in rural or secluded areas as access to electricity can be limited. Coupling these systems with photovoltaic (PV) cells can resolve this issue.^[^ ^27^, ^38^, ^56^ ^]^ Additionally, current state‐of‐the‐art PV cells currently available are capable of achieving only 5–20% efficiency in real‐world conditions. This decrease in efficiency is primarily attributed to the rise in surface temperatures, exposing the cell to operate constantly under high temperatures.^[^ ^72^ ^]^ BR distillation modes can utilize this thermal by‐product for water evaporation in a multistage configuration as shown schematically in **Figure** **9**.^[^ ^13^, ^73^ ^]^ Simultaneous solar cell cooling and high freshwater production to optimize energy and freshwater production is one way to promote further practicality to existing designs. For example, using a PV‐membrane distillation‐evaporator (PME) can reduce the overall temperature of the solar cell beyond 10 °C.^[^ ^11^ ^]^

As examined above, multi‐stage membrane distillation and vapour generation systems still require high temperatures to utilize for the distillation processes. The limitation of these systems is that they rely on the total heat production by photothermal or PV materials. Photovoltaic thermal (PVT) technologies may use gas or liquid‐based heat transfer fluids to generate high thermal energy from the solar absorber or PV cells.^[^ ^74^ ^]^ PVT collectors that concentrate and harvest the thermal energy produced by the PV cells are limited to ≈80 °C.^[^ ^74^ ^]^ When considering these high‐performing PVT collectors, it is important to consider the effects of high temperatures on the residue from the brine water. The foulants remaining from the distillation process, such as calcium carbonate, magnesium hydroxide, and calcium phosphate, have an inverse solubility with temperature.^[^ ^74^, ^75^ ^]^

Considering the limitations of BR distillation modes, the passive inflow of brine water from the capillary wick and evaporators is one of the main restricting factors for scalability and freshwater production. The co‐production of electricity and water opens the possibility of using mechanical devices for increased mass flow and low voltage in photothermal‐electrothermal hybrid systems. Controlling the flow rate within these systems allows for ease in optimizing the freshwater production rate and overcoming the limit of evaporation area scalability, as mechanical power is necessary for inlet flow to cover the total evaporation area in the initial stage for only a short period of time to achieve full saturation. However, additional mechanical devices increase cost, energy consumption, complexity of operation, maintenance, and scalability as these modular systems are focused on practical implementation in rural, secluded regions and areas in crisis. Although passive flow does not require external power sources and significantly reduces the cost of the device, it is faced with shortcomings in long‐term use and total surface area scalability. Currently, passive systems are limited due to their inability to create capillary action across a large surface area and face issues with uniformity and consistency. When utilizing the energy generated from the PV cell for hybrid electrothermal processes, the generated electricity needs to be stored in batteries for it to be used during low sunlight or night‐time operation. This also adds to the cost and overall complexity of the system. A mobile power supply unit has demonstrated sufficient ultra‐high evaporation results as discussed in the above section,^[^ ^50^ ^]^ and solar cells plus batteries were used in a multistage distillation system, achieving remarkable outcomes in terms of sustained long‐term performance and the system's self‐sufficiency in energy and freshwater production.^[^ ^56^ ^]^

## 4 Challenges and Perspectives

**Figure 10:**
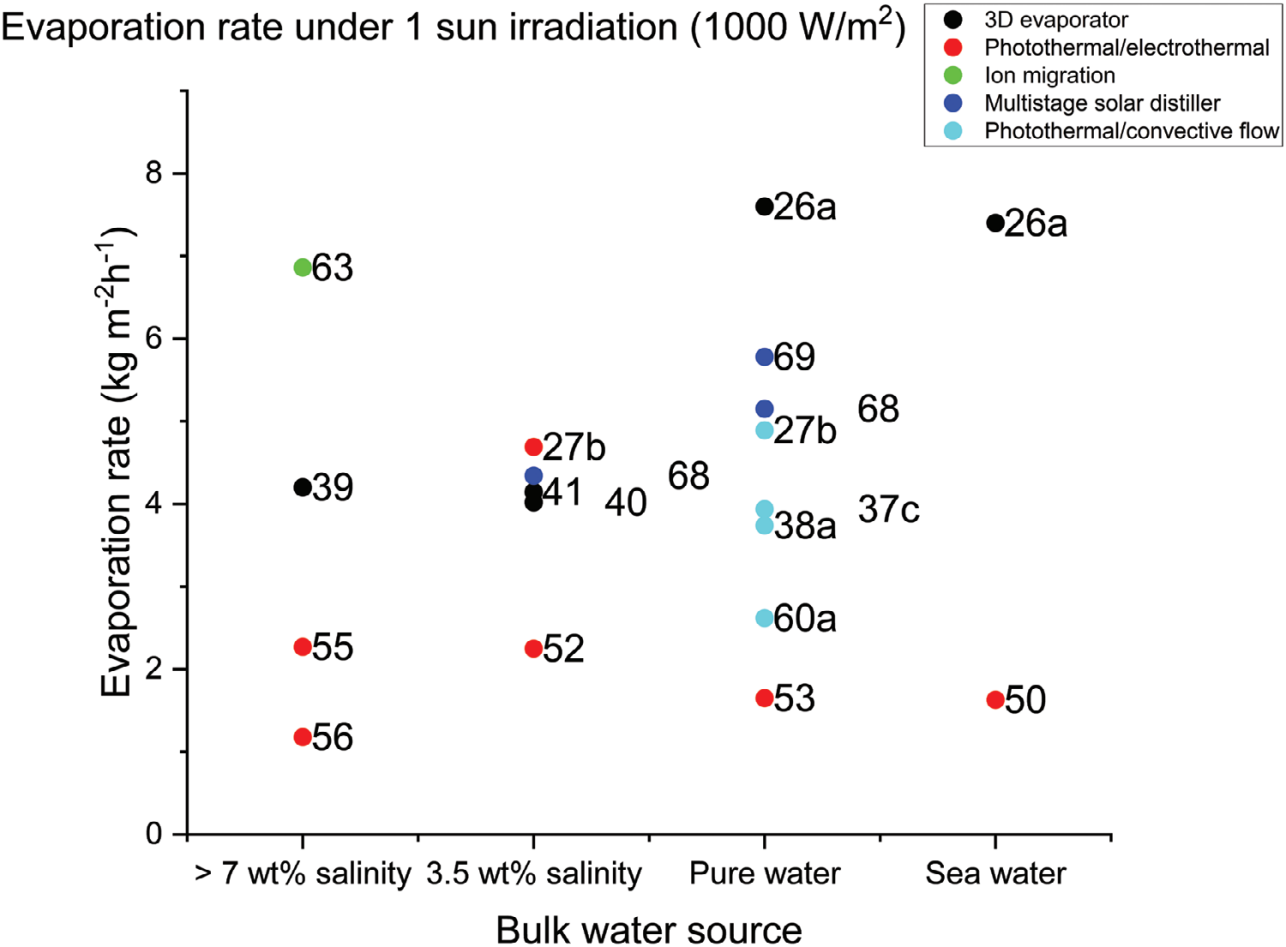
Graphical representation of evaporation rates of various SDIE designs reviewed in this study under 1 Sun irradiation.

In this review, novel and innovative designs for SDIE with high rates have been explored. The designs have utilized secondary energy sources such as environmental energy (dark energy), electrothermal conversion and wind to enhance the water evaporation rate. It is evident that reliance solely on solar energy has limitations, especially in outdoor environments where weather conditions and time of day alters the total solar energy available. Designs with SDIE coupled with convective flow can exhibit evaporation rates more than double those of 3D evaporators solely reliant on solar energy. When harnessing energy sources such as solar, ambient temperature, and wind resources, it is essential that the rate of pure water evaporation reaches a level that is practical and applicable for its intended purpose. The exploration of designs with evaporation rates above 4 kg m^−2^ h^−1^ as summarized in **Table** **1** and graphically presented in **Figure** **10** have displayed potential for practically applicable designs according to their use of energy resources available.

**Table 1:** Comparison of designs of high evaporation rate SDIE devices and performances in literature.

Design type	Evaporation rate [kg m^−2 ^h^−1^]	Energy conversion efficiency	Energy input	Bulk water source	Design description	Cost	References
3D evaporator	7.6	178%	1 Sun	Pure water	3D reduced graphene oxide; sodium alginate coated cotton hollow (14 cm height, 2.5 cm diameter)		[26a]
	7.4		1 Sun	Sea water			
3D evaporator	4.02	220%	1 Sun	3.5 wt.% salinity	3D volcanic shaped polydopamine sprayed polylactic acid evaporator (1.5 cm height, 5 mm top diameter, 15 mm bottom diameter)		[40]
3D evaporator	4.2	179%	1 Sun	14 wt.% salinity	3D corrugated Fe‐doped carbon nanotubes (5.5 cm tall foam water channel)		[39]
3D evaporator	4.145	120%	1 Sun	3.5 wt.% salinity	3D PPy/alginate/poly(n‐isopropylacrylamide) cylindrical hydrogel (4 cm height)		[41]
3D evaporator	4.32	282.4%	1 Sun	Pure water	3D heatsink‐like spray‐coated porous nanocarbon composites bamboo sheet paper		[26b]
Photothermal/Electrothermal	11.7		1 Sun, 5V	Yellow Sea seawater	3D zeolite‐chitosan‐TiO2@PPy aerogel		[50]
	5.72		5V				
	1.63		1 Sun				
Photothermal/Electrothermal	1.65	98%	1 Sun	Pure water	Carbon back nanoparticle coated copper oxide needle array etched onto copper foam		[53]
	4.5		2V				
Photothermal/Electrothermal	6.73		1 Sun, 2V	3.5 wt.% salinity	3D all‐carbon conductive foam (1 cm thickness)		[52]
	2.25	87%	1 Sun				
Photothermal/Electrothermal	2.54		1 Sun	Pure water	Reduced flexible rGO mixed carbon cloth		[54]
	26.52		1 Sun, 3V				
Photothermal/Electrothermal	9.02	287%	1 Sun, 10 V	7 wt.% salinity	Melamine sponge supported reduced graphene oxide	$11.315 m^−2^	[55]
	2.27		1 Sun				
Photothermal/Electrothermal	10.5		0.5 Sun, 36V	10 wt% salinity	Sandwich structure MXene‐graphene oxide‐Mxene on polyvinylidene fluoride membrane		[57]
	1.18		1 Sun				
	2.12		0.5 Sun, 5V				
Photothermal/Convective flow	28.4		1 Sun, 6 ms^−1^	3.5 wt.% salinity	Vertical black cloths (4.3 × 2 cm^2) lined in nylon frame		[60b]
Photothermal/Convective flow	11.9		1 Sun, 4 ms^−1^	Pure water	CSC spray coated cloth wrapped around metal frame (4.2 cm diameter, total evaporation surface 108 cm^2^)		[37c]
	3.938		1 Sun				
Photothermal/Convective flow	2.62	97%	1 Sun	Pure water	Facile extrusion‐based 3D printed pyramid shaped electro spun polyacrylonitrile/carbon nanotubes	$6.7 m^−2^	[60a]
	8.31		1 Sun, 4 ms^−1^				
Photothermal/Convective flow	4.89	125%	1 Sun	Pure water	3D hybrid reduced graphene oxide and polyvinyl alcohol ester (3 cm thickness)	$40 m^−2^	[27b]
	16.22		1 Sun, 2.5 ms^−1^				
	4.69		1 Sun	3.5 wt.% salinity			
	15.41		1 Sun, 2.5 ms^−1^				
Photothermal/Convective flow	3.74		1 Sun	Pure water	3D printed bacterial cellulose, sodium alginate cross‐linked glutaraldehyde carbon nano powder aerogel (3.5 × 3.5 mm^2^ matrix size, 4 cm height)		[38a]
	25.3		1 Sun, 2 ms^−1^				
Multistage solar distiller	5.78	385%	1 Sun	Pure water	10 stage thermally localised solar still (solar absorbing are 9.6 × 9.6 cm^2^)	$1.54 per unit (10 × 10 cm^2^ unit)	[69]
Multistage solar distiller	5.15		1 Sun	Pure water	8 stage flexible passive multistage membrane distillation – only considering dead end mode		[68]
	4.34		1 Sun	3.5 wt.% salinity			
Ion migration/Hydrogel	6.86		1 Sun	15 wt.% salinity	Janus cationic anionic hydrogel		[63]

Numerous advantages and practical applications of ultra‐high SDIE and vapor generation technologies are apparent, however some challenges remain as follows:

1) Direct comparison of the merits of various systems is problematic as they may harness and require different energy sources at different levels.

Figure 10 displays the evaporation rates of different designs under 1 Sun irradiation. It is evident that photothermal coupled with joule heating displays the lowest performance under 1 Sun irradiation. This may be due to the use of 2D evaporator designs and is limited to conductive materials, limiting the energy drawn from the environment. On the other hand, SDIE coupled with convective flow and those involving 3D design where environmental energy can be harnessed, show higher evaporation rates as the increase in surface area and exposure of the porous medium allows for increased vapour escape out of the evaporator. To optimize for each design purpose, when applying to a practically applicable design or modular system, the implementation strategies are significantly different as they require different energy sources. When a condenser is needed to serve the purpose of freshwater collection, it directly hinders with solar absorption, access to environmental energy and convective flow. Moreover, the location of the evaporation site associates with the amount of environmental, wind and solar energy available. Many of the 3D evaporators have shown efficiencies beyond 100% due to their ability to harness environmental energy. As of now, the method of calculating energy to vapour conversion efficiency has not been standardized as the consideration of enthalpy of an evaporative material has been determined through three main methods: DSC analysis, Clausius‐Clapeyron equation or constant latent heat of vaporisation at 100 °C.^[^ ^10^, ^76^ ^]^ It is stated that utilizing the constant latent heat of vaporization at specific temperatures overestimates the conversion efficiency.^[^ ^77^ ^]^ As seen in Table 1, the conversion efficiency value is not always provided and is under various energy inputs while evaporating different bulk water sources.

2) The balance of techno‐economic benefits of utilizing coupled/secondary energy sources.

For most SDIE evaporators, the sole reliance on solar energy can limit the duration of operation and stability. In most cases, the designs purely reliant on solar energy require certain geometric and microstructural parameters to draw out maximum efficiency from the material making it difficult for upscaling and practical application. Thus, alternative energy sources have been explored to address these limitations. However, it is possible that the inclusion of the photothermal element on the designs adds no techno‐economic benefit to the overall evaporation rate. Enhancing a functioning water evaporator with a photothermal component, while offering only marginal improvements, might not be a cost‐effective or eco‐friendly addition to the system. As seen in Figure 10, photothermal coupled with electrothermal systems displayed the lowest evaporation rates between 1.5 – 2.5 kg m^−2 ^h^−1^ under 1 Sun irradiance. This range is relatively high compared to other 2D SDIE systems but comparatively lower when compared to 3D or other design systems. There is a need to re‐evaluate the cost implications when integrating the photothermal element and examining its technical advantages added to the overall system performance and the quality of the produced water. The financial and environmental aspects must be considered as additional layers of materials, especially non‐biodegradable materials, which will affect sustainability and economic issues. Some recommendations include the use of an overall sustainable footprint (OSF) analysis with the following equation:^[^ ^50^, ^52^, ^78^ ^]^

(5) $$\mathrm{OSF}=100\%\times \sum_{j\;=\;1}^{8}{\left(\frac{1}{3}\right)}_{j}\times \frac{1}{8}$$

where j represents the chosen eight sustainability factors, namely: resources, cost, preparation simplicity, recyclability, evaporation rate, removal rate, thermal management, and environmental friendliness, and i represents the score for each factor from Low (i = 1), Medium (i = 2), and High (i = 3). Out of the chosen factors, an increase in cost is a negative factor; therefore Low (i = 3) and High (i = 1). The energy‐to‐vapour conversion efficiency can be used in correspondence or included in the factors as the calculation method becomes more standardised.

3) Gaps between evaporation rates and water collection rates (water production)

The purpose of design is important as well as its limits when coupled with different energy sources as the achievement of ultra‐high evaporation rate can become unproductive. The parameters of evaporation rate, solar to vapour conversion efficiency and freshwater collection should be clearly distinguished.^[^ ^21^ ^]^ The interconnection between the evaporators and distillers lies in their practical applications, primarily focusing on vapor generation. However, a clear separation between usable freshwater collection and concentration of bulk water exists. Although the gap between evaporation and vapor generation is a single step away from the condensation process, it is imperative to consider this step, especially when the evaporator might be employed for freshwater production. SDIE systems coupled with convective flow are prone to this factor as when applied in practical environments for water collection, multi‐directional access to wind is very limited as the condenser needs to cover a portion of the system.^[^ ^27b^ ^]^ Achieving an ultra‐high evaporation rate is an attractive element in terms of harnessing passive solar or thermal energy, as it holds significant potential for practical applications. Still, it does not directly address the primary goal of ensuring access to freshwater for human use. Other resource recovery applications involve soil remediation,^[^ ^65^, ^79^ ^]^ salt production^[^ ^80^ ^]^ and resource recovery from industry wastewaters.^[^ ^81^ ^]^

In outdoor conditions, SDIE will be exposed to harsh and fluctuating environmental conditions, where the reliability and stability of the performance will be hindered by physical damage and instability in energy input associated with its practicality. Until both these major drawbacks are resolved, it is not feasible for these SDIE systems to be deployed for industrial purposes. On the other hand, multistage systems explored in this review for freshwater production displays potential in practical application as they are able to produce satisfactory levels of water (above 4 kg m^−2^ h^−1^).^[^ ^68^, ^69^ ^]^ Although, the challenge for upscaling is still present, most of the materials used in these devices are cheap and accessible.

4) Fouling and scaling issues

Some universal challenges, such as fouling and scaling issues, still have not been fully addressed. For long‐term practical application, anti‐fouling methods for seawater sources, including both biofouling and, crystallization should be addressed.^[^ ^28a^ ^]^ Figure 10 reveals a lack of experimental data with higher salinity bulk water and photothermal convective flow systems mostly testing with pure water sources when tested under 1 Sun irradiation. This is a critical area of research, as ultimately these evaporators will be employed as desalination, wastewater evaporation or purification methods. Although high performance is evident, resistance to fouling and scaling will be a key factor when employed in practical environments. Long‐term stability in some cases may be more appealing in comparison to high evaporation performance as the total volume of water evaporated or freshwater production may exceed in the long run. This can be advantageous for communities requiring reliable freshwater production and in some industrial applications where the rate of evaporation is not important. Extensive examination of prolonged operational periods remains relatively unexplored in the existing literature, primarily due to the prevailing trend of testing evaporative systems for relatively short durations of 6 to 8 h. However, achieving stable, continuous operation over the course of days and even weeks offers significant advantages. It leads to reduced maintenance requirements, lower operational costs, and an overall enhancement in evaporation or water production efficiency.

Some salt management strategies have been introduced, such as diffusion, ion‐migration strategies, directional salt crystallization and surface dilution strategies.^[^ ^64^ ^]^ Regarding the ultra‐high evaporation designs above, hydrogels typically utilize backflow or diffusion methods to crystallize salts toward the edges of the photothermal surface. While strategies for managing salt have been discussed, the capacity to completely prevent salt accumulation and crystallization over prolonged durations still presents an unanswered question.^[^ ^82^ ^]^ Furthermore, addressing the methods for cleaning and replacing specific materials within the evaporative layer in the event of salt build‐up is a topic that requires further attention to ensure practicality in the long term.

5) Self‐reliance and adaptability

Self‐reliance is a definite merit for coupled systems needing external energy input. From the application perspective, access to alternative/secondary energy sources can be restricted to locations where electricity and high wind speeds are obtainable. Adopting self‐reliance, especially in freshwater production, poses significant benefits as operational needs are reduced and can simplify matters at the user end. Photo‐voltaic cells have been used to generate the power needed in electrothermal processes.^[^ ^50^, ^55^, ^74^ ^]^ Photothermal and electricity storage methods may be an effective way to resolve this issue for stable all day evaporation. In addition, adaptability of these systems should be considered in cases where the photothermal material is faced away from the sun or a shade appears above the material. In these cases, the evaporation rate will be significantly reduced.

6) Upscaling size for practicality

In the current SDIE technology, laboratory scale samples cannot fully represent large scale applications as there are many limitations of upscaling the size and variations in operational effects across different outdoor environments.^[^ ^8^, ^66^ ^]^ Upscaling the size of SDIE systems for freshwater production and industry scale is a significant challenge, as passive high evaporation rate materials mostly require an intricate fabrication process and joule heating systems will need a significant increase in electrical energy input based on total surface area. Additionally, depending on the geometrical shape of 3D evaporator design,^[^ ^83^ ^]^ in cases where the heat transfer is needed for vaporization in the evaporator, upscaling the size for practical usage may result in a significant reduction in the initial evaporation rate from both reduced heat transfer and mass flow rate. When looking at the capillary rise in vertical systems, the capillary action is directly hindered by gravitational acceleration as the water is transported perpendicular to the water's surface. The capillary action equation is represented as:

(6) $$h=\frac{2\gamma \cos\propto }{\rho_{w}gr}$$

where *γ* is the surface tension of water, α is the contact angle, *ρ_w_* is the density of water, *g* is the gravitational acceleration to the direction of water flux, and *r* is the size of pores in the porous medium.

In evaporator designs where vertical increase in the geometric shape imposes significant improvements in the evaporation rate, the maximum height of capillary action will need to be considered during the upscaling phase. As there is an inverse relationship between permeability and capillary pressure,^[^ ^14^ ^]^ the increase in maximum height of capillary action will hinder the vapor flux through the evaporator medium. This is directly associated to 3D evaporator designs as 2D systems often require a shorter total distance for vapor to pass through during evaporation. For horizontal wicks, capillary action is associated to the pore size of the transport medium. As the surface area increases, the capillary action is determined on the consistencies in the porous medium ultimately determining the stability of water transport across the evaporator. This is an area to consider when examining upscaling for practical applications.

## 5 Conclusion

SDIE is a promising technology to address issues regarding access to clean water and passive methods of water evaporation. The designs and approaches of SDIE toward high evaporation are rich and varied. The benefits are unequivocal, as they have demonstrated practically sound results. However, there are still significant areas to evaluate when considering ultra‐high interfacial solar water evaporation technologies. Ultimately, regardless of their purpose, the discussed research above has managed to produce high levels of evaporation and purified water in accordance with standardized health and safety standards.

The practicality of SDIEs with high evaporation rates is highly desirable in terms of benefits over other high energy‐consuming alternatives for desalination, water purification and pollutant/resource concentrations, especially in remote and resource‐limited areas. However, the technology is still in its development stage as standardized water collection methods have not been delved into deeply, as well as maintenance of high light transmittance through humid and vapour‐dense conditions under vapour collection techniques. Most of the reported high evaporation performances are still based in laboratory settings, and the real, outside‐world practical application is yet to be fully implemented. For industrial use, most materials face durability issues and the inability to operate for long‐term periods, involving fouling issues on evaporators from pollutant and salt accumulation. To consider water purification and freshwater generation from complex water bodies, SDIE systems are yet to delve further into how they manage wastewater and highly polluted seawater. Modifications to consider organic pollutants will require pre‐treatment, advanced oxidation, and adsorption techniques for clean water production. The insights of this review surface the current front liners in producing high evaporation rates in SDIE and report the progress made by both passive and systems with alternative secondary energy inputs.

## Conflict of Interest

The authors declare no conflict of interest.
